# Radical hysterectomy in the elderly

**DOI:** 10.1186/1477-7819-6-38

**Published:** 2008-04-07

**Authors:** Azamsadat Mousavi, Mojgan Karimi Zarchi, Mitra Modares Gilani, Nadereh Behtash, Fatemeh Ghaemmaghami, Maryam Shams, Maryam Irvanipoor

**Affiliations:** 1 Gynecologic Oncology Department, Vali-e-Asr Hospital, Imam Khomeini Hospital Complex, Keshavarz Blvd., Tehran 14914, Iran

## Abstract

**Background:**

The considerable increase in life expectancy on one hand and an increase in cervical cancer among Iranian patients on the other, brings out the importance of investigating whether radical surgery can be performed safely and effectively on patients above 60 years of age.

**Methods:**

In a study of historical cohort, all 22 patients 60 years and above who have undergone a Wertheim radical hysterectomy for cervical cancer from 1999 to 2005 were compared with 128 matched cases under 60 years of age who had undergone a Wertheim hysterectomy during the same calendar year. All patients were analyzed for preexisting medical comorbidities, length of postoperative stay, morbidity, and postoperative mortality.

**Results:**

There was no operative mortality in either group, morbidity (minor, p = 0.91; major, p = 0.89) were statistically not different in the two groups despite the patient's above 60 years having significantly higher comorbidity prior to surgery than the younger cohort (minor, *P *< 0.05; major, *P *< 0.05). The mean postoperative hospital stay was significantly longer in the older patients (5 days vs. 3 days, *P *< 0.001).

**Conclusion:**

Wertheim Radical hysterectomy is a safe surgical procedure in the selected population of patients 60 years and over. No differences in operative mortality or morbidity were found when compared to a cohort of patient's aged 60 years or younger.

## Background

Uterine cervical cancer is the most common neoplasia of primary gynecological malignant disease in many countries; In Iran after breast cancer, cervical cancer is the second most common female cancer and according to a hospital-based registry, the incidence of this malignancy is 6–8/100000; moreover life expectancy is increasing and so is the percentage of elderly patients. Despite the fact that surgery and radiotherapy are equally effective for treatment of early cervical cancers, the latter is less preferable due to unexpected complications and long-term consequences [1,2].

Radical hysterectomy involves the removal of cervix, uterus, and supporting tissues, with the pelvic lymphadenectomy and removal of upper third vagina [1-4].

Although 5-year survival is more than 90% for node negative disease [2], the procedure conveys significant morbidity and it is important to know whether radical surgery can be performed on elderly patients with negligible morbidity. Surgical morbidity encountered in elderly patients after radical hysterectomy has previously been addressed in the literature; radical hysterectomy has been reported as a safe surgical procedure in patients 60 years and over [3-5].

The purpose of this study was to investigate the morbidity and mortality of radical hysterectomy in the elderly patients (defined as those 60 years or over) and generalize the information disseminated in the previous report by incorporating a central group of non-elderly patients (defined as those less than 60 years).

## Methods

The hospital records were reviewed on all patients who underwent Wertheim radical hysterectomy and pelvic lymphadenectomy for FIGO stage IB-IIA cervical cancer form March 1999 to December 2005[4]. Operations were all performed by 4 consultant gynecologic oncologists who have the same experience in this field. This work was approved by Tehran University of medical science review board.

In all cases general anesthesia with endotheraceal intubations was used. Induction was carried out with short-acting barbiturate followed by the use of inhaled anesthetic agents. Postoperatively nasogastric decompression was not routinely used.

The morbidity rates, preexisting medical problems, postoperatively mortality rates, and length of postoperative hospitalization were compared with a randomly selected series of non-elderly patients who had undergone a similar surgery during the same calendar year.

Minor preoperative morbidity such as history of angina without any recent problems and/or not being on any daily medication and minor complications consisted of hypertension, poor nutritional preoperative status, obesity, thyroid disease and neurological disorders such as multiple sclerosis. Obesity was defined as being at least 20% overweight for height and age based upon the increased health risk shown for this level by the Framingham study [6]. Major preexisting medical problems included sever arteriosclerotic heart disease, angina, with a history of cerebrovascular accidents or transient ischemic attacks, insulin-dependent diabetes mellitus, a history of past pulmonary embolism and moderate to sever chronic obstructive lung disease. The significance of preexisting medical conditions was determined by the fellow or gynecologic resident involved in the care of the specific patient and problem.

Minor surgical morbidities were: temperature elevations of 100.4 F or higher on two separate occasions six hours apart and more than 24 h post surgery, pneumonia, urinary tract infections, inadvertent cystotomies, adynamic ileus, lymphocyte formation, and wound infections. Major surgical morbidities were: myocardial infarctions, other significant cardiac events, cerebrovascular accidents, small bowel obstructions, urethral or bladder fistula, urethral injury, deep vein thrombosis, wound evisceration, and any complication requiring secondary, major invasive surgery. In general major surgical morbidity seemed to lengthen hospital stay more than 3 days. Bladder dysfunction was defined as the need for either continuous drainage or intermittent catheterization (self or otherwise). Long-term bladder dysfunction was defined as bladder dysfunction lasting greater than or equal to 3 months.

Radical hysterectomy was defined as removal of the uterus, cervix, at least the upper third of the vagina, the division of the uterosacral ligaments at their point of insertion pararectally, the division of the cardinal ligaments at their origin on the obtrator fosa, the complete unroofing of the lower portion of ureters and removal of all tissue lateral to the ureters, the most lateral margin being the pelvic wall. All pelvic lymphadenectomies were therapeutic in nature including (bilaterally) the following nodal groups: external iliac, internal iliac, obtrator, and common iliac to the bifurcation of the aorta.

### Statistical analysis

Students't test was used to compare mean postoperative hospital stay between two groups and chi-square test for comparing mortality and morbidity between the groups. Significant level was set at 0.05 and the analysis was carried out using SPSS version 11.5.

## Results

Twenty-two elderly women aged were identified to have undergone a Wertheim radical hysterectomy with pelvic lymphadenectomy for FIGO stage IB (IB1-1B2) and IIA cervical cancer. Patients were randomly matched with 6 non-elderly patients operated on in the same year. The second most common preexisting condition found in both groups was hypertension (Table 1). The most common major comorbidities in both groups were diabetes mellitus, ischemic heart disease and hyperlipidemia. Over all, both minor and major preexisting comorbidities were more common in the elderly patients (p, 0.05)(Tables 1 and 2). In fact 68.4% of elderly patients versus 29.6% of non-elderly patients had at least one comorbidity.

**Table 1:** Preexisting comorbidities

Preexisting conditions	≤ 60 Years old	>60 years old
	Number(%)	Number(%)

Ischemic heart disease	7(0.6)	2(8.7)
Hypertension	9(7.2)	5(23.5)
Diabetes mellitus (I or II)	8(6.2)	2(8.7)
Chronic obstructive lung disease	0	1(4.5)
Peripheral vascular disease	1(0.7)	1(4.5)
Thyroid disease	2(1.7)	1(4.5)
Previous pelvic radiation	9(7.2)	3(13)

**Table 2:** Intraoperative and postoperative complications

**Preexisting conditions**	**≤ 60 Years old**	**>60 years old**
	Number(%)	Number(%)

Prolonged ileus	25(20.1)	1(4.5)
Cystitis/cystotomy	0	0
Pneumonia	0	0
Urethral injury	3(2.7)	0
Incontinence	3(2.7)	0
Lymphocyte formation	0	0
Deep vein thrombosis/embolism	0	0
Wound infection	12(9.3)	2(9)
Bowel obstruction	0	0
ICU admission	0	0
Fever	10(7.8)	4(18)

The most common intraoperative complication in both groups was hemorrhage (27.3% in elderly patients versus 39.1% in non-elderly). This could be due to history of previous abdominal surgery which was more common in younger patients. The most common postoperative complication in both groups was prolonged adynamic ileus whereas in the older age group, pulmonary emboli were more common (2 patients in older age group versus no one in younger group). Duration of bladder catheterization in older patients was longer than younger's (13.45 ± 1.8 days vs. 11.34 ± 4.6 days). There was no significant difference in postoperative morbidity (minor, P = 0.91; major, P = 0.89) between the younger and older cohorts. None of the patients in either group had long-term bladder dysfunction.

Considering the entire time period of the study, the group of elderly patients due to higher comorbidity had longer mean hospital stay than the other age group. For each time period analyzed, the older age group stayed on average two more days in the hospital compared to the younger age group (5 versus 3 days).

## Discussion

During the recent two decades, life expectancy has been increasing from 68 to 78 in Iran. The introduction of cervical screening programs in the developed world has resulted in a reduction in the incidence of cervical cancer as well as in the earlier detection of the disease [1-3]. Unfortunately in developing countries, advanced cervical cancer is still one of the most common cancers.

Baranovsky and Mayers demonstrated that the incidence of cervical cancer in elderly women (65 years or more) is 1.2 times that of patients aged 45–64 years [7]. Therefore, the question of how to treat older patients' malignancy becomes of considerable challenge.

Seeking for a reasonable answer, the authors decided to analyze whether a Wertheim hysterectomy could be safely performed in the population of 60 years or older. Similarly Geisler and Geisler examined the morbidity and surgical mortality from radical hysterectomy in elderly patients (65 years of age and older), which was not a case-control study [5]. Later they compared results of Wertheim in two populations: above 50 and below 50 [2]. The present study was a continuation to their study, with restriction to patients with IA2 and IB cervical cancer, and an additional cohort of younger patients matched by year of surgery with the study group. Although the younger cohort had less comorbidity, there was no significant increase in operative or postoperative complications found. Younger patients did, however, have significantly shorter mean hospital stay [2].

Previous authors have compared the morbidity and mortality of radical hysterectomy in elderly (age 65 years or older) patients versus younger patients (age 64 years or less) in developed countries [8-11]. In the current study, Iranian patients 60 years and over were compared to those under 60 in order to determine whether geographic location would affect the previous results. Although Fuchtner *et al*., and Kinney *et al*., recruited younger patients in their cohort [8,9]; they did not find any differences in morbidity between these age groups.

Based on our findings mortality and morbidity in elderly patients undergoing Wertheim hysterectomy is quite negligible comparing to younger patients. Therefore patients with the mentioned criteria seem to have no restriction undergoing such surgeries.

## Conclusion

Wertheim Radical hysterectomy is a safe surgical procedure in the selected population of patients 60 years and over. No differences in operative mortality or morbidity were found when compared to a cohort of patient's aged 60 years or younger.

## Competing interests

The author(s) declare that they have no competing interests.

## Authors' contributions

MAZ and KZM: wrote this article and edited the manuscript, SM and IM: conception and editing of manuscript, MMG, NB, and FG conducted the literature search, and helped in preparation of manuscript.

All authors read and approved the final manuscript.
